# Cardiovascular risk prediction models for women: A systematic review protocol

**DOI:** 10.1371/journal.pone.0360860

**Published:** 2026-10-15

**Authors:** Cairina Frank, Hadi Tehfe, Charles Ghanem, Jade Choo-Foo, Ramtin Hakimjavadi, Hawre Jalal, Risa Shorr, Daniel Kobewka

**Affiliations:** 1 Department of Medicine, The Ottawa Hospital, Ottawa, Ontario, Canada; 2 The University of Ottawa, Ottawa, Ontario, Canada; 3 Library Services, The Ottawa Hospital, Ottawa, Ontario, Canada; 4 Bruyere Research Institute, Ottawa, Ontario, Canada; Athens Medical Group, Psychiko Clinic, GREECE

## Abstract

**Purpose:**

Cardiovascular disease remains a leading cause of morbidity and mortality in women. Cardiovascular risk prediction models are widely used to guide preventative management, although many do not incorporate female-specific risk factors. The aim of this review is to identify published cardiovascular risk prediction models for women or those which include female-specific predictors and to summarize the predictors and model performance.

**Methods:**

The protocol was developed following the Preferred Reporting Items for Systematic Reviews and Meta-Analyses (PRISMA-P) guidelines. The protocol has been registered in PROSPERO (CRD420261420766). An update of the systematic review by Baart et al. will be performed by searching MEDLINE and EMBASE from July 27 2017 to May 2026 to identify primary articles reporting on the development and/or validation of models predicting incident cardiovascular disease in women. Data on model predictors, performance, validation and clinical usability will be extracted. Risk of bias will be assessed using PROBAST+AI. Findings will be synthesized descriptively although quantitative synthesis will be considered when sufficiently comparable studies are available.

**Conclusions:**

This systematic review will provide an updated synthesis of cardiovascular risk prediction models in women and summarize how female-specific risk factors are incorporated into contemporary risk assessment.

## Background

Cardiovascular disease (CVD) remains the leading cause of morbidity and mortality [1]. It is estimated that up to 75% of premature CVD is preventable if early risk factor identification and modification is offered [1–3]. Well-established sex differences exist in CVD, including variability in clinical symptoms, pathophysiology and prognosis [4–6]. Notably, women experience inferior outcomes after cardiac events possibly due to under-representation in research and lack of consideration of sex-specific risk enhancers in clinical assessment, highlighting the importance of improving cardiovascular risk recognition in this population [7].

Cardiovascular risk prediction models are an important component of primary prevention and inform decisions regarding management and intensity of follow-up. However, their clinical utility depends on accurate risk estimation in the populations to which they are applied [8]. While many traditional risk factors are shared between sexes, there are several well established female-specific variables known to contribute to increased CVD risk including early menopause as well as pregnancy complications such as hypertensive disorders of pregnancy or preterm birth [9–10]. Studies suggest that general population risk estimates may not fully capture cardiovascular risk in young women, particularly in those with a history of adverse pregnancy outcomes (APO) [11]. Recognizing APOs as significant risk factors for future cardiovascular disease allows for timely identification and engagement in preventative strategies, particularly during the postpartum period when individuals are well connected to the healthcare system. Even modest risk reductions, when applied early in individuals with a long remaining life expectancy, can accrue over time and translate to meaningful reductions in cardiovascular events [12].

Over the past two decades, numerous prediction models have been developed to identify individuals at high risk for CVD and to help inform clinical decisions. Despite increasing recognition of sex-specific risk enhancers, many widely used models remain uniform between sexes and do not incorporate female specific predictors [13]. Determining whether female-specific predictors provide incremental prognostic value beyond established risk factors may further provide opportunities to improve care. Baart et al. conducted a systematic review of cardiovascular risk prediction models for women with the literature search concluding in 2017. Since that time, recognition of female-specific risk factors has increased alongside an emergence in model development and thus an updated review is warranted to evaluate contemporary evidence and examine the incremental predictive value of female-specific risk factors.

The aim of this review is to identify and characterize cardiovascular risk prediction models in women and evaluate the incremental value of female-specific predictors. We will summarize the model predictors, validation, performance and clinical usability for prediction of fatal and non-fatal cardiovascular outcomes.

## Methods

We will perform a systematic review in accordance with the Preferred Reporting Items for Systematic Reviews and Meta-Analyses (PRISMA-P) guidelines (S1 Table). The protocol is registered on PROSPERO (ID: CRD420261420766). Any important amendments to this protocol will be updated in PROSPERO and described in the final publication. This review received no funding and had no sponsor and thus no funder or sponsor had any role in the design of the protocol.

This review will update the search by Baart et al published in 2019. The present review will include only studies identified by the updated search period and will not incorporate studies included in the original Baart et al review [13]. We will search Medline and EMBASE from July 27 2017 until May 2026 to identify primary articles reporting the development and/or validation of models predicting incident cardiovascular disease in women. As this review is designed as an update of Baart et al., we will retain the same databases used in the original review to ensure continuity of the search strategy while maintaining broad coverage of the biomedical and cardiovascular literature.

### Eligibility criteria

We will include articles which report on at least one multivariable prediction model, tool or score for individual risk estimation of future CVD outcome in women. Models will be included if they report on: 1) models developed in female-only cohorts, 2) general population models in which sex is included as a predictor or as an effect modifier, 3) studies that externally validate existing models (validation papers) in female-only or sex-stratified populations and 4) studies that investigate the added value of female-specific predictors to existing models (incremental value papers) including but not limited to: premature menopause or menarche, polycystic ovary syndrome, pre-eclampsia, gestational diabetes, gestational hypertension, preterm delivery, fetal growth restriction, endometriosis or perinatal depression. Prediction models using statistical methods, machine learning or artificial-intelligence approaches will be eligible if they meet all other inclusion criteria. While resulting in a broader review, general population models will be included if they explicitly incorporate sex as a predictor or effect modifier, or if they are validated in a female-only or sex-stratified population), as these models provide relevant information on how sex is incorporated into cardiovascular risk estimation and how established models perform when applied specifically to women.

Eligible studies will be classified into the following groups:

1. Development studies of CVD prediction models in female-only cohorts (will be defined as female specific models)
2. Development studies of CVD prediction models in general population but include sex as predictor or effect modifier (will be defined as sex predictor or sex modifier models)
3. Validation studies where performance of an existing model is verified in female only or sex-stratified populations (will be defined as validation papers)
4. Incremental value studies which evaluate the addition of female-specific predictors to existing CVD risk models (will be defined as incremental value papers)

Articles will be included if they report models for predicting any fatal or non-fatal arterial CVD event including coronary heart disease, myocardial infarction, stroke, cardiovascular death or congestive heart failure. Articles must be in English or French and report original research. We will exclude articles describing models for predicting risk of venous disease, validation articles with a cross sectional study design or articles describing models developed from or validated exclusively in specific diseased populations such as patients with diabetes, chronic kidney disease or HIV. However, studies conducted in female-specific or obstetric populations (such as those with hypertensive disorders of pregnancy) will remain eligible for inclusion.

### Information sources

The search strategy from Baart et al will be updated in collaboration with a medical librarian (S1 Text). We will perform an update of their search using Medline and EMBASE from July 27 2017 until May 2026 to identify primary articles reporting on development and/or validation of models predicting incident cardiovascular disease in women. Letters, conference abstracts and conference presentations will be excluded. Reference lists of included studies will be screened to identify any additional eligible articles. No additional electronic databases, trial registries or contact with study authors are planned for this review.

### Study records

Study records will be managed with Covidence Systematic Review software. Articles will be reviewed independently and in duplicate by two authors based on title and abstract followed by full text. Disagreements will be resolved through group consensus.

### Data extraction

Data will be extracted into a pre-specified spreadsheet based on the Checklist for critical Appraisal and data extraction for systematic Reviews of prediction Modelling Studies (CHARMS) [14]. Data extraction will be done independently by one author and independently verified by a second author. Disagreements will be resolved by group consensus. Where required information is not reported, data will be recorded as unavailable. We will extract information regarding model development and validation, sample size and number of outcome events. Further information to be extracted includes study design, study population, geographic location, outcome definition, prediction, modelling method, number and type of predictors, methods for handling missing data, handling of data, internal and external validation procedures, model performance measures (discrimination, calibration and reclassification) and measures of clinical usability (number of predictors, availability of model equation and online calculator).

For included incremental value papers, we will also extract information on the model that was used and which predictors had incremental value. For those describing external validation, we will also include which model was validated, type of validation and performance metrics.

### Risk of bias

Risk of bias assessment will be completed using the Prediction model Risk of Bias Assessment Tool and Artificial Intelligence (PROBAST+AI) [15]. Assessments will be completed independently by one reviewer and checked by a second reviewer with disagreements resolved through group consensus. Risk of bias assessments will be used to inform the interpretation of study findings and overall strength of the evidence.

### Study status

The literature search will include studies published between July 27 2017 and May 2026. Early screening of titles/abstracts has been started at time of submission. We anticipate completion of literature screening, data extraction and risk of bias assessment by January 2027 with data synthesis and manuscript preparation completed by May 2027.

### Data synthesis

Results will be summarized using descriptive statistics. Quantitative synthesis will be considered only where at least three external validation studies evaluate the same prediction model using comparable outcomes, prediction horizon and performance measures. The availability of three studies will be considered a minimum criteria and not in itself sufficient for meta-analysis. Where appropriate, pooled c-statistics as well as calibration measures (calibration intercept and slope) will be estimated using random-effects meta-analysis. C-statistics will be transformed to the logit scale prior to pooling. Standard errors will be obtained from reported data. Pooled estimates and corresponding 95% confidence intervals will subsequently be back-transformed to original c-statistic scale for presentation. Statistical heterogeneity will be assessed using the I^2^ and τ^2^ and a 95% prediction interval will be reported when possible. Where multiple eligible performance estimates are reported from the same study population, the estimate that most corresponds to the outcome and prediction horizon being evaluated in that meta-analysis will be selected. Pooled estimates will be interpreted cautiously given expected differences in populations, outcome definitions and follow-up duration. Where sufficient studies are available, sensitivity analyses based on risk of bias assessments will be considered including analysis excluding studies judged to have significant risk of bias. Where quantitative synthesis is not appropriate, findings will be synthesized narratively by comparing model characteristics, predictors, model performance and measures of clinical usability. Analyses will be performed in R.

Formal assessment of publication bias is not planned as quantitative synthesis is anticipated only for a limited number of external validation studies. No formal certainty of evidence framework will be applied. The strength of the evidence will consider clinical and methodological heterogeneity and risk of bias assessment.

## Discussion

This systematic review will present an updated synthesis of cardiovascular risk prediction models with particular attention to model performance and the incremental value of female-specific risk factors. This review will aim to clarify whether contemporary practices adequately capture cardiovascular risk prediction in women and whether incorporation of female-specific factors improves model performance.

The review has been designed in accordance with established guidance for systematic reviews of prediction models. Study selection will be performed independently and in duplicate, data extraction will follow the CHARMS framework and risk of bias will be systematically assessed using PROBAST+AI. Where studies are sufficiently comparable, quantitative synthesis will be considered, otherwise, model characteristics will be synthesized narratively. This review is relevant given increasing recognition that several female-specific factors, including adverse pregnancy outcomes, are associated with cardiovascular disease. However, the extent to which these factors contribute additional predictive information beyond traditional risk factors remains uncertain.

This study was designed as an update of Baart et al. and as such maintains continuity with the previous search strategy by only searching MEDLINE and EMBASE. While these databases provide broad coverage, this restriction may result in eligible studies being missed and thus reference lists of included studies will be screened to mitigate this limitation. Heterogeneity in study populations, prediction horizons and reported measures of model performance may also limit feasibility of quantitative synthesis.

## Conclusion

This systematic review will provide an updated analysis of the current evidence on cardiovascular risk prediction models for women and clarify whether the inclusion of female-specific risk factors provides predictive value. These findings may inform future model development and identify priorities for research.

## Supporting information

S1 Table PRISMA-P Checklist. (DOCX)

S1 Text Example search strategy for MEDLINE. (DOCX)
