# Supplementary material for: Cardiovascular risk prediction models for women: A systematic review protocol
Source: PLoS One. 2026 Oct 15;21(10):e0360860. doi: 10.1371/journal.pone.0360860 (PMC13671513; doi:10.1371/journal.pone.0360860)
Supplement: Supplementary file 1 [file pone.0360860.s001.docx]

**S1 Table – PRISMA-P Checklist**

| **Section & Topic** | **Item No.** | **Checklist Item** | **Page #** |
| --- | --- | --- | --- |
| **Title** Identification Update | 1a 1b | Identify report as protocol of a systematic review If the protocol is an update of a previous systematic review, identify as such | 1 5 |
| **Registration** | 2 | If registered, provide the name of the registry and registration number | 4 |
| **Authors** Contact Contributions | 3a 3b | Provide name, affiliation, e-mail address of all authors, provide mailing address of corresponding Describe contributions of protocol authors and identify the guarantor of the review | 1 1 |
| **Amendments** | 4 | If the protocol represents an amendment of a previously completed or published protocol, identify as such and list changes; otherwise, state plan for documenting important protocol amendments | 4-5 |
| **Support** Sources Sponsor Role of Sponsor | 5a 5b 5c | Indicate sources of financial or other support for the review  Provide name for the review funder and/or sponsor  Describe roles of funder(s), sponsor(s) and/or institution(s), if any, in developing the protocol | 5 5 5 |
| **Rationale** | 6 | Describe the rationale for the review in the context of what is already known | 3-4 |
| **Objectives** | 7 | Provide an explicit statement of the question(s) the review will address with reference to participants, interventions, comparators and outcomes (PICO) | 4 |
| **Eligibility Criteria** | 8 | Specify the study characteristics (such as PICO, study design, setting, time frame) and report characteristics (such as years considered, language, publication status) to be used as criteria for eligibility for the review | 5-6 |
| **Information sources** | 9 | Describe all intended information sources (such as electronic databases, contact with study authors, trial registers or other grey literature sources) with planned dates of coverage | 6-7 |
| **Search strategy** | 10 | Present draft of search strategy to be used for at least one electronic database, including planned limits, such that it could be repeated | 16-21 |
| **Study Records** Data management Selection process Data collection process | 11a 11b 11c | Describe the mechanism that will be used to manage records and data throughout the review  State the process that will be used for selecting studies through each phase of the review  Describe planned method of extracting data from reports, any processes for obtaining data | 7 7 7 |
| **Data items** | 12 | List and define all variables for which data will be sought (such as PICO items, funding sources) and pre-planned data assumptions and simplifications | 7-8 |
| **Outcomes** | 13 | List and define all outcomes for which data will be sought, with rationale | 6 |
| **Risk of bias** | 14 | Describe anticipated methods for assessing risk of bias of individual studies, including whether this will be done at the outcome or study level, or both; state how this information will be used in data synthesis | 8 |
| **Data synthesis** | 15a 15b 15c 15d | Describe criteria under which study data will be quantitatively synthesised Describe planned summary measures, methods of handling data and combining data  Describe any proposed additional analyses  If quantitative synthesis is not appropriate, describe type of summary planned | 8-9 8-9 8-9 8-9 |
| **Meta-biases** | 16 | Specific any planned assessment of meta-biases | 8-9 |
| **Confidence in evidence** | 17 | Describe how the strength of the body of evidence will be assessed | 9 |
