# Supplementary material for: Cardiovascular risk prediction models for women: A systematic review protocol
Source: PLoS One. 2026 Oct 15;21(10):e0360860. doi: 10.1371/journal.pone.0360860 (PMC13671513; doi:10.1371/journal.pone.0360860)
Supplement: Supplementary file 2 [file pone.0360860.s002.docx]

**S1 Text – Example search strategy for MEDLINE**

Ovid MEDLINE(R) ALL <1946 to May 19, 2026>

1	chd risk assessment$.mp.	103

2	cvd risk assessment$.mp.	648

3	heart disease risk assessment$.mp.	73

4	coronary disease risk assessment$.mp.	6

5	cardiovascular disease risk assessment$.mp.	286

6	cardiovascular risk assessment$.mp.	2535

7	cv risk assessment$.mp.	215

8	cardiovascular disease$ risk assessment$.mp.	293

9	coronary risk assessment$.mp.	90

10	coronary risk scor$.mp.	95

11	heart disease risk scor$.mp.	81

12	chd risk scor$.mp.	115

13	cardiovascular risk scor$.mp.	1095

14	cardiovascular disease$ risk scor$.mp.	293

15	cvd risk scor$.mp.	628

16	cv risk scor$.mp.	145

17	or/1-16	5826

18	cardiovascular diseases/	201445

19	coronary disease/	134180

20	cardiovascular disease$.mp.	391539

21	heart disease$.mp.	309704

22	coronary disease$.mp.	144639

23	cardiovascular risk?.mp.	102369

24	coronary risk?.mp.	6224

25	exp hypertension/	338780

26	exp hyperlipidemia/	73777

27	or/18-26	1124379

28	risk function.mp.	496

29	Risk Assessment/mt	48094

30	risk functions.mp.	424

31	risk equation$.mp.	1115

32	risk chart?.mp.	372

33	(risk adj3 tool$).mp.	33716

34	risk assessment function?.mp.	12

35	risk assessor.mp.	68

36	risk appraisal$.mp.	864

37	risk calculation$.mp.	1320

38	risk calculator$.mp.	3058

39	risk factor$ calculator$.mp.	7

40	risk factor$ calculation$.mp.	18

41	risk engine$.mp.	274

42	risk equation$.mp.	1115

43	risk table$.mp.	247

44	risk threshold$.mp.	2596

45	risk disc?.mp.	5

46	risk disk?.mp.	15

47	risk scoring method?.mp.	82

48	scoring scheme?.mp.	1076

49	risk scoring system?.mp.	1591

50	risk prediction?.mp.	29207

51	predictive instrument?.mp.	203

52	project$ risk?.mp.	325

53	cdss.mp.	3536

54	or/28-53	116360

55	27 and 54	15749

56	17 or 55	19994

57	new zealand chart$.mp.	3

58	sheffield table$.mp.	35

59	procam.mp.	278

60	General Rule to Enable Atheroma Treatment.mp.	2

61	dundee guideline$.mp.	1

62	shaper scor$.mp.	0

63	(brhs adj3 score$).mp.	3

64	(brhs adj3 risk$).mp.	5

65	copenhagen risk.mp.	6

66	precard.mp.	5

67	(framingham adj1 (function or functions)).mp.	58

68	(framingham adj2 risk).mp.	4726

69	framingham equation.mp.	173

70	framingham model$.mp.	138

71	(busselton adj2 risk$).mp.	1

72	(busselton adj2 score$).mp.	1

73	erica risk score$.mp.	1

74	framingham scor$.mp.	616

75	dundee scor$.mp.	3

76	brhs scor$.mp.	3

77	British Regional Heart study risk scor$.mp.	1

78	brhs risk scor$.mp.	2

79	dundee risk scor$.mp.	4

80	framingham guideline$.mp.	3

81	framingham risk?.mp.	3942

82	new zealand table$.mp.	6

83	ncep guideline?.mp.	180

84	smac guideline?.mp.	2

85	copenhagen risk?.mp.	6

86	or/57-85	5792

87	56 or 86	23864

88	exp decision support techniques/	86852

89	Decision Support Systems, Clinical/	11235

90	Diagnosis, Computer-Assisted/	25334

91	algorithms/	350957

92	algorithm?.mp.	652953

93	algorythm?.mp.	102

94	decision support?.mp.	59750

95	predictive model?.mp.	49642

96	treatment decision?.mp.	38316

97	scoring method$.mp.	5651

98	(prediction$ adj3 method$).mp.	19012

99	or/88-98	866471

100	Risk Factors/	1083423

101	exp Risk Assessment/	349991

102	(risk? adj1 assess$).mp.	442129

103	risk factor?.mp.	1615601

104	100 or 101 or 102 or 103	1912132

105	27 and 99 and 104	8150

106	87 or 105	29549

107	stroke.mp.	448687

108	exp Stroke/	200643

109	cerebrovascular.mp. or exp Cerebrovascular Circulation/	168835

110	107 or 108 or 109	591868

111	110 and 54	7500

112	110 and 99 and 104	3965

113	111 or 112	10643

114	106 or 113	36552

115	(women or female*).tw,kf.	2624010

116	Sex Factors/	295623

117	(sex or gender).tw,kf.	1254662

118	female/	10654017

119	Sex Characteristics/	66514

120	115 or 116 or 117 or 118 or 119	11475900

121	114 and 120	22810

122	limit 121 to dt=20170727-20260519	11746
